# Visuospatial Attention Allocation as an Indicator of Cognitive Deficit in Traumatic Brain Injury: A Systematic Review and Meta-Analysis

**DOI:** 10.3389/fnhum.2021.675376

**Published:** 2021-07-20

**Authors:** Jacinta A. Walz, Revathy Mani, Mohammed M. Alnawmasi, Sieu K. Khuu

**Affiliations:** School of Optometry and Vision Science, The University of New South Wales, Sydney, NSW, Australia

**Keywords:** visual attention, endogenous, exogenous, visual cue, traumatic brain injury

## Abstract

Traumatic Brain Injury (TBI) is defined by changes in brain function resulting from external forces acting on the brain and is typically characterized by a host of physiological and functional changes such as cognitive deficits including attention problems. In the present study, we focused on the effect of TBI on the ability to allocate attention in vision (i.e., the use of endogenous and exogenous visual cues) by systematically reviewing previous literature on the topic. We conducted quantitative synthesis of 16 selected studies of visual attention following TBI, calculating 80 effect size estimates. The combined effect size was large (g = 0.79, *p* < 0.0001) with medium heterogeneity (I^2^ = 68.39%). Subgroup analyses revealed an increase in deficit with moderate-to-severe and severe TBI as compared to mild TBI [*F*_(2, 76)_ = 24.14, *p* < 0.0001]. Task type was another key source of variability and subgroup analyses indicated that higher order attention processes were severely affected by TBI [*F*_(2, 77)_ = 5.66, *p* = 0.0051). Meta-regression analyses revealed significant improvement in visual attention deficit with time [p(mild) = 0.031, p(moderate-to-severe) = 0.002, p(severe) < 0.0001]. Taken together, these results demonstrate that visual attention is affected by TBI and that regular assessment of visual attention, using a systematic attention allocation task, may provide a useful clinical measure of cognitive impairment and change after TBI.

## Introduction

Traumatic Brain Injury (TBI) can be defined as changes in brain function, or other brain pathology, caused by external forces acting on the brain (Menon et al., 2010). Alterations in brain function commonly associated with TBI include loss of consciousness, loss of memory known as post-traumatic amnesia, disorientation, and other changes in mental state (Kay et al., 1993; Langlois et al., 2006; Jagnoor and Cameron, 2014; Pervez et al., 2018). TBI may occur due to falls, hits, motor vehicle accidents, blasts such as those incurred in armed combat or sports-related injuries (Menon et al., 2010; Jagnoor and Cameron, 2014; Pervez et al., 2018; James et al., 2019). As a result, TBI affects all areas of the population and particularly, males, children aged zero to 4 years, and adolescents aged 15 to 19 years are more likely to sustain injury (Langlois et al., 2006; Jagnoor and Cameron, 2014; Nguyen et al., 2016). This is likely due to mobility problems in young children, risk-taking behavior in new drivers and increased activity in adult males and adolescents as falls and motor-vehicle accidents are among the leading causes of TBI (Hyder et al., 2007; Jagnoor and Cameron, 2014; Nguyen et al., 2016).

A pressing public health problem with an annual incidence of 349 cases per 100,000 globally, (Nguyen et al., 2016). TBI cases result in over 200,000 hospitalisations in the United States alone (Langlois et al., 2006) and are a leading cause of death and disability (Jagnoor and Cameron, 2014; Nguyen et al., 2016; James et al., 2019). In addition to long term disability, significant economic burden and reduced quality of life are often incurred as a result of injury (Hyder et al., 2007; James et al., 2019).

TBI is typically graded as mild, moderate, or severe using diagnostic and prognostic tools such as the Glasgow Coma Scale, which assess the degree of injury based on loss of consciousness, memory loss, or patient responses to different levels of stimuli (Reith et al., 2016). Mild TBI accounts for 70–90% of TBI cases (Nguyen et al., 2016; James et al., 2019), although the long-term impact of mild TBI is significantly lower than that of moderate or severe injury, with the majority of post-injury symptoms resolving within 3 months to a year, while more severe injuries may be symptomatic throughout life (Eisenberg et al., 2014; Hiploylee et al., 2017). However, grading TBI in three broad categories does not adequately capture different archetypes of TBI as the extent of deficit across key outcome measures is likely to be a continuum in scale. Accordingly, current and the lack of standard conventions in the classification of TBI may not be entirely appropriate to grade the scope and scale of deficits that are associated with TBI.

The impact of TBI on daily lives of patients is often crippling. Post-Concussion Syndrome is the name given to a whole host of physical, emotional, and cognitive symptoms commonly experienced following TBI. Commonly reported somatic symptoms include headaches, fatigue, and dizziness which, especially when chronic, may interfere with the individual's ability to navigate their daily lives (Vanderploeg et al., 2007; Eisenberg et al., 2014). Emotional control centers are often affected in TBI resulting in outbursts of anger and uncontrollable mood swings which may impact patients and put pressure on their relationships with family and friends (Vanderploeg et al., 2007; Gorgoraptis et al., 2019).

Cognitive processing issues, including memory and attention deficits, are commonly reported and have been well-investigated (Binder et al., 1997; Belanger et al., 2005; Frencham et al., 2005; Vanderploeg et al., 2007; Mani et al., 2018). A key meta-analysis assessing the evidence of cognitive deficit and neuropsychological performance following mild TBI was conducted by Binder et al. (1997). This review assessed a wide range of neuropsychological functions in patients suffering TBI and studies were included if patients had a positive history of mild TBI at least 3 months prior, regardless of the presence of symptoms. The overall effect size, weighted for sample size, was small (g = 0.07) however there is contention regarding whether this result was influenced by their study selection process. The authors only included studies with participants with a positive history of TBI and excluded those studies where participants were recruited based on clinical presentation or referral for symptom management. As a result, studies of symptomatic TBI patients were not accounted for in the review and it is unclear whether the effect described is similar, or larger in a symptomatic population. When further investigation was conducted and studies of symptomatic TBI populations were included, overall effect size for neuropsychological outcome increased (Belanger et al., 2005) as did effect sizes for specific cognitive domains (Zakzanis et al., 1999).

Binder et al. (1997) also calculated effect sizes for specific cognitive domains. The only area which indicated significant cognitive deficit was attention (Hedges' g = 0.17), which are general and complex processes to selectively take notice of specific information in the environment (Wickens et al., 2003). Clearly, cognitive processing, and in particular attention, is impacted in TBI of all severities, however, the full extent of deficit remains at present unclear and the focus of much research (McCrea et al., 2009; Mani et al., 2018, 2020; Snegireva et al., 2018; Walz et al., 2020).

The visual system has long been used to index attention processing as it provides a simple and non-invasive means of assessing a variety of cognitive function (Posner and Petersen, 1990; Petersen and Posner, 2012). Visual attention is the ability to selectively focus on specific elements of *visual* information (Posner et al., 1980; McMains and Kastner, 2009). This distinction is considered semantic as the cortical areas recruited during attention processing are common mechanisms despite being applied to different sensory domains (Klingberg, 1998; Adcock et al., 2000; Bunge et al., 2000; Macaluso, 2006; Nijboer et al., 2014; Moisala et al., 2015). Assessments of visual attention following TBI report conflicting evidence of the nature of this deficit (Cremona-Meteyard et al., 1992; Cremona-Meteyard and Geffen, 1994b; Hills and Geldmacher, 1998; Van Donkelaar et al., 2005; Halterman et al., 2006; Pavlovskaya et al., 2007; Catena et al., 2009; Schmitter-Edgecombe and Robertson, 2015). It is likely that the heterogeneity associated with this effect indicates that visual attention is not a single process, but rather a more complex, multi-stage aspect of cognition that is affected inconsistently by TBI across different domains.

For several decades eye movements and pupil responses have been investigated using different types of eye-tracking technology as surrogate measures of attention (van der Wel and van Steenbergen, 2018; Hunt et al., 2019). In addition to behavioral evidence, there is strong neurological support for the link between the major attention processing networks and the neural systems responsible for eye movements (Eckstein et al., 2017) and pupil responses (Daniels et al., 2012; Wang and Munoz, 2015). Furthermore, eye movements and pupil responses have been used to identify deficits or altered attention processing in a number of diseased populations including TBI. While eye movements have been particularly well-investigated, (Mani et al., 2018) altered pupil responses as a marker for attention following TBI have only recently been identified (Walz et al., 2020) and require further investigation as a potential biomarker for attention deficit.

With further investigation into the specific nature of attentional deficit is required, many researchers have turned to visual search paradigms for evidence. Visual search tasks provide a unique opportunity to assess baseline visuospatial attention capacity (Treisman and Gelade, 1980). Whilst paradigms may differ, the general premise of identifying target shapes, letters, or figures amongst distractors in visual space remains a strong basis for assessing the ability to scan visual space for pertinent information (Treisman and Gelade, 1980; McElree and Carrasco, 1999).

Geldmacher and Hills conducted two studies assessing visual search capacity following severe TBI (Geldmacher and Hills, 1997; Hills and Geldmacher, 1998). Using simple cancellation visual search tasks, they identified poorer performance by the TBI population. This effect was amplified in more attention-demanding tasks where the target-to-distractor ratio resulted in increased task difficulty, i.e., target-to-distractor ratio was 1:9 instead of 1:4 (Geldmacher and Hills, 1997). It is unclear whether this effect was the result of increased response time or poorer accuracy as they reported a “Q score” which combines both effects as a product of the proportion of correct responses dependent on completion time and the total number of targets.

An increase in search load resulting in poor task performance suggests a deficit in the internal task-driven attention system, as opposed to attention driven by salience in the visual field. This notion of a higher-order deficit is further supported by Schmitter-Edgecombe and Robertson (2015) who reported significantly slowed visual search rates in the moderate-to-severe TBI population when target salience was reduced. These two responses, conventionally requiring endogenous and exogenous processes, are the primary drivers of visual attention allocation. Typically, an endogenous response uses task-specific information to drive visual search for relevant information while the exogenous response utilizes visual scene properties, such as salience, to capture visual attention.

Commonly, these systems are assessed using visual search tasks and cue-response tasks such as the Covert Orienting of Attention Task (Posner, 1980; Posner and Cohen, 1984; Posner and Petersen, 1990), and Attention Network Test (Fan et al., 2005). The Covert Orienting of Attention Task assesses the endogenous system by capturing visual attention with directional cues while the Attention Network Test assesses exogenous attention by engaging the bottom-up response with peripheral spatial cues. These directional and spatial cues are considered *valid* when the target appears in the cued location, and *invalid* when the target appears elsewhere (Posner, 1980; Posner and Cohen, 1984). Healthy individuals exhibit a faster response time or benefit with a valid cue when compared with a no cue condition. Conversely, an invalid cue results in an increased response time or cost due to the need to reorient spatial attention to the target location (Posner, 1980; Posner et al., 1980).

Cremona-Meteyard et al. (1992) and Cremona-Meteyard and Geffen (1994b) assessed endogenous orienting of attention using the Covert Orienting of Attention Task in individuals who had sustained a closed head injury at 2-weeks, 1-year, and more than 1-year post-injury. They identified persistent, slowed attention allocation processing in both mild and moderate-to-severe brain injury patients, consistent with increased latency event-related potentials and attenuated cortical responses (Cremona-Meteyard and Geffen, 1994a). Further, head injury patients showed reduced benefit in reaction time from a correct directional cue and increased costs in response to an incorrect cue, indicating decreased capacity to allocate attention, and disengage and reallocate to new information when relying on an endogenous response.

On the other hand, Van Donkelaar et al. (2005) investigated exogenous attention allocation using the Attention Network Test and reported slowed processing immediately following injury. This was evidenced by uniform delayed reaction times across all task conditions. Furthermore, the addition of an exogenous spatial cue to a typical visual search task improved reaction time in TBI patients to a greater degree than controls, suggesting that the bottom-up stimulus driven response remains predominantly intact when compared with the higher order endogenous response deficit following TBI.

Unlike the persistent deficits in endogenous attention allocation identified by Cremona-Meteyard and Geffen (1994b) on further investigation, the altered exogenous processing reported by Van Donkelaar and others improved to recovery at just 1-month post-injury (Halterman et al., 2006) In particular, the increased reaction time benefit exhibited by TBI patients when using the spatial cue was not evident at any subsequent testing date in the month following injury, despite persistent slowed reaction times overall. It should be noted that only one group has investigated the disengagement and reorienting process for exogenous attention allocation following TBI (Pavlovskaya et al., 2007) and one other, only at the preliminary pilot study level (Sinnett et al., 2011).

Within the literature, there is much disparity regarding the specific nature of these deficits, including the extent of deficit, the areas and severity groups affected, persistence and recovery, and the influence of discrepancies in task design. To date, there has been no comprehensive systematic assessment/review of *attention allocation capacity* in the TBI population. In order to gain understanding of the nature and degree of attention allocation and processing deficits following TBI, a systematic review of the relevant literature was conducted to qualitatively assess the discrepancies in task designs, recruitment processes, and severity groups. A meta-analysis of the data included in this literature was used to identify and quantify deficits in attention processing and allocation following TBI. Further, the relationships between this effect and task type, injury severity, age, time since injury and outcome measure were investigated using subgroup and meta-regression analyses.

## Materials and Methods

This study was conducted according to the PRISMA guidelines for reporting systematic review and meta-analysis (Page et al., 2021). This review protocol has been registered with PROSPERO (CRD42020199419).

### Search Strategy

A literature search was performed on the NLM PubMed, Cochrane Library, and Google Scholar databases aiming to retrieve relevant articles that investigated visual attention in all severities of TBI. The search was performed using the search strategy described in Table 1. The search was conducted from May to September 2020. In order to avoid missing relevant literature, a backward and forward search of eligible articles was performed. The backward search was conducted from the reference list of eligible studies and forward search was performed from the list of articles that cited the eligible studies included in the review.

**Table 1:** Search strategy used in PubMed database.

1 Traumatic brain injury or TBI
2 Head injury
3 Spatial attention
4 Visuospatial attention
5 Attention orienting
6 Covert Orienting of Attention
7 Attention Network Test
8 Visual search
9 1 or 2
10 3 or 4 or 5 or 6 or 7 or 8
11 9 and 10
12 Limit 11 to yr = “2020”

### Study Selection

Studies were included if they met the “PICOS” principles.

1) Population: Studies of human adults (aged 18 years and over) with validated assessment and recruitment processes were included. Non-human studies, and human studies of children and adolescents were excluded.

2) Intervention: Studies that assessed human subjects with at least one episode of head injury with no intervention.

3) Comparison: Studies that had a comparable control group were included in terms of age and gender. Those adult studies were excluded if they did not include a relevant control group or if they implemented an active treatment plan for recovery from TBI. These could be partially included with adequate baseline data; however, follow-up data was not included. Further, if an article did not report task specific results, e.g., reporting a single average response time despite having both cued and un-cued tasks included in the assessment, then first authors were contacted for raw data. If the necessary data was provided, the article was included, else it was excluded from the meta-analysis but retained for quantitative analysis.

4) Outcome: Outcome measures that assessed visual attention allocation such as response time of correct trials, Studies that reported data in mean and standard deviation were included. Those that reported otherwise were only included where conversions were possible, e.g., standard error to standard deviation conversion. If measures were reported graphically, WebPlotDigitizer (Rohatgi, 2015) was used to extract the relevant information and the necessary conversion were conducted as needed.

5) Study design: Only case-control studies included in this review. Case reports, case series, studies that had irrelevant task design, outcome measures, and/or with intervention were excluded.

### Data Extraction

Two independent reviewers (JW and RM) screened abstracts against the inclusion and exclusion criteria (see Table 2). A consensus was required before a study could be included in the review. If JW and RM disagreed on the eligibility of a study, a third reviewer (SK) was involved and an agreement about its inclusion reached after group discussion. The basis of these criteria was to ensure that study designs were similar in the way they assessed visual attention following TBI and to isolate those studies that provided adequate data for the calculation of the necessary effect sizes.

**Table 2:** Inclusion and exclusion criteria.

**Inclusion criteria**	**Exclusion criteria**
English Language or English Language translation available	Non-English studies without available English translations
Extractable data; Results reported as mean and standard deviation or standard error of the meanCase-control study design with matched controls	Case reports, reviews; irrelevant study designs including active treatment plans and no control group
Graded TBI^*a*^ of adults (aged 18 and over)	Unextractable data; data reported as median and range
Reported TBI factors (such as etiology and time since injury)	Studies of children and adolescents (aged under 18 years)
Task design included visual search, Covert Orienting of Attention Task, Attention Network Test or another valid cueing spatial search paradigm	Studies where outcome measures are not task specific, e.g., imaging measures
Key outcome measures are specific to task performance e.g., task accuracy, reaction time	

a *Traumatic brain injury*.

Each article was assessed for risk of bias using critical appraisal tools provided by the Jonna Briggs Institute System (JBI) for case-control studies. Using this tool, the methodological quality of each study was analyzed to determine the extent to which it addressed the possibility of bias in its design, conduct and analysis (Moola et al., 2017). Table 3 reports the risk of bias assessment for individual study included in the review. The reported in this table the great majority of studies met the checklist requirements for case-control studies and therefore the risk of bias is low. Only three studies (papers) were assessed as being unclear or “no” on certain items on the checklist. Note that all studies adopted a between groups design (controls vs. TBI) and so the requirement for equal exposure was not applicable as controls did not have TBI. Given this assessment we are confident that the risk of bias in our metanalysis is low.

**Table 3:** Assessment of risk of bias.

**Author and year**	**Were the groups comparable other than the presence of disease in cases or the absence of disease in controls?**	**Were cases and controls matched appropriately?**	**Were the same criteria used for identification of cases and controls?**	**Was exposure measured in a standard, valid and reliable way?**	**Was exposure measured in the same way for cases and controls?**	**Were confounding factors identified?**	**Were strategies to deal with confounding factors stated?**	**Were outcomes assessed in a standard, valid and reliable way for cases and controls?**	**Was the exposure period of interest long enough to be meaningful?**	**Was appropriate statistical analysis used?**
Hills and Geldmacher (1998)	Yes	Yes	Yes	Yes	NA	Yes	No	Yes	Yes	Yes
Bate et al. (2001)	Yes	Yes	Yes	Yes	NA	Yes	Yes	Yes	Yes	Yes
Halterman et al. (2006)	Yes	Yes	Yes	Yes	NA	Yes	Yes	Yes	Yes	Yes
Pavlovskaya et al. (2007)	Yes	Yes	Yes	Yes	NA	Yes	Yes	Yes	Yes	Yes
Kim et al. (2009)	Yes	Yes	Yes	Yes	NA	Yes	Yes	Yes	Yes	Yes
Hill-Jarrett et al. (2015)	Yes	Yes	Yes	Yes	NA	Yes	Yes	Yes	Yes	Yes
Cremona-Meteyard et al. (1992)	Yes	Yes	Yes	Yes	NA	Yes	Yes	Yes	Yes	Yes
Cremona-Meteyard and Geffen (1994b)	Yes	Yes	Yes	Yes	NA	Yes	Yes	Yes	Yes	Yes
Macflynn et al. (1984)	Yes	Yes	Yes	Yes	NA	Yes	Yes	Yes	Yes	Yes
Geldmacher and Hills (1997)	Yes	Yes	Yes	Yes	NA	Yes	Yes	Yes	Yes	Yes
Van Donkelaar et al. (2005)	Yes	Yes	Yes	Yes	NA	Yes	Yes	Yes	Yes	Yes
Catena et al. (2009)	Yes	Yes	Yes	Yes	NA	Yes	Yes	Yes	Yes	Yes
Sinnett et al. (2011)	Yes	Yes	Yes	No	NA	Yes	Yes	Yes	Yes	Yes
Rodríguez-Bailón et al. (2012)	Yes	Yes	Yes	Unclear	NA	Yes	Yes	Yes	NA	Yes
Schmitter-Edgecombe and Robertson (2015)	Yes	Yes	Yes	Yes	NA	Yes	Yes	Yes	Yes	Yes
Robertson and Schmitter-Edgecombe (2017)	Yes	Yes	Yes	Yes	NA	Yes	No	Yes	Yes	Yes
Shah et al. (2017)	Yes	Yes	Yes	Yes	NA	Yes	Yes	Yes	Yes	Yes
Hromas et al. (2020)	Yes	Yes	Yes	Yes	NA	Yes	Yes	Yes	Yes	Yes

“*Yes”, Low risk of bias; No, high risk of bias; NA, Not applicable*.

Articles that met the inclusion criteria had relevant outcome and key measures extracted and recorded in Microsoft Excel. The extracted data included TBI etiology, severity and grading, post-injury period, task design information, sample sizes of TBI and control groups, participant ages, and relevant performance measures including response time, task accuracy, and other reported scores relevant to task performance, including mean and standard deviations as appropriate. Each study was compared across based on classification of TBI according to the severity indices, study design, post-injury period, attention task design, sample size, nature of population and outcome measures investigated.

The mean and SD of outcome measures from case and controls were used to conduct a meta-analysis. A meta-analysis is a statistical procedure used to consolidate the existing evidence of the relationship between a number of factors. Meta-analyses provide a unique opportunity to mathematically assess the overall relationship between these factors by detecting trends across multiple studies which a single sample study may fail to highlight.

The majority of included studies reported more than one condition, either by varying task design or repeating the same task at multiple time points or in different samples. As such, multiple effect sizes were calculated for each study, as was necessary. Hedges' *g* effect size was calculated to determine the relationship between TBI and control group task performance. For the majority of studies, which reported response time as the key outcome measure, a positive effect size indicated a poorer performance by the TBI group, i.e., slower response time, while a negative effect size indicated a better performance by the TBI group than the control group (see Equation 1).

Few studies, on the other hand, reported task accuracy or another measure which indicated a poorer performance by the TBI group with a negative effect size. In these instances, all of which reported a negative value, the absolute value of the Hedges' *g* effect size was calculated in order to keep in line with the other reported group relationships.

Hedges' *g* effect size was calculated by taking the difference in the mean outcome measure of the TBI case and control groups and dividing this difference by the pooled standard deviation of the study populations. This can be represented by the formula:

(1) $$\begin{array}{r} Hedges\;ES=g=\frac{M_{1}-M_{2}}{Pooled\;SD} \end{array}$$

Where *M*_1_ and *M*_2_ represent the means of the TBI cases and the control groups, respectively. The pooled standard deviation was calculated using the following formula:

(2) $$\begin{array}{r} Pooled\;SD=\;\sqrt{\frac{\left(N_{1}-1\right)SD_{1}^{2}+\;\left(N_{2}-1\right)SD_{2}^{2}}{N_{1}+\;N_{2}-2}} \end{array}$$

Where *N*_1_ and *N*_2_ represent the respective sample sizes of the case and control groups and, similarly, *SD*_1_ and *SD*_2_ refer to the standard deviations of each of the case and control group scores, respectively. The 95% confidence intervals of Hedges' *g* were calculated by *g* ± 1.96*SD*_*g*_ where *SD*_*g*_ is calculated by

(3) $$\begin{array}{r} SD_{g}=\;\sqrt{\frac{\left(N_{1}+\;N_{2}\right)}{N_{1}.N_{2}}\;+\;\frac{g^{2}}{2\left(N_{1}+\;N_{2}\right)}} \end{array}$$

Hedges' *g* effect size, *SD*_*g*_, and 95% confidence intervals were calculated for each relevant task performance measure in the included studies.

An absolute effect size value for Hedges' *g* of < 0.3 would be considered a small effect size, while a value of 0.3–0.5 was considered moderate, and an effect size of >0.5 was considered to reflect a large difference between the compared groups (Hedges, 1981).

### Statistical Analysis

Extracted data were entered into Microsoft Excel using the *Meta-Essentials v1.4* workbook (Suurmond et al., 2017) and all figures were generated using GraphPad Prism 8. A measure of heterogeneity was generated by assuming a random effect model and utilizing the Inverse Variance method (Borenstein et al., 2009). The *Q* statistic and *I*^2^ index were calculated to assess the variability and heterogeneity of the effect sizes in the meta-analysis.

The *Q* statistic null hypothesis indicates homogeneity in the sample size-weighted effect sizes. In this instance, a chi-squared distribution and *k-1* degrees of freedom are assumed, where *k* indicates the number of effect sizes included. If the *Q* statistic is found to be significant the null hypothesis of homogeneity is rejected, and a random effect model can be applied including within- and between-studies variability measures.

The *I*^2^ index was calculated in order to provide a measure of the degree of heterogeneity as the *Q* statistic only indicates statistical significance of heterogeneity (Huedo-Medina et al., 2006). *I*^2^ was calculated using the following formula:

(4) $$\begin{array}{r} I^{2}=\;\frac{Q-df}{Q}.100 \end{array}$$

Where *Q* refers to the heterogeneity value, and *df* is the degrees of freedom. The *I*^2^ index gives a percentage value from 0 to 100 where low, medium, and high heterogeneity can be approximated by *I*^2^ values of 25, 50, and 75% respectively (Huedo-Medina et al., 2006).

Hedges' *g* effect size was calculated for each appropriate outcome measure in every accepted article. As a result, most studies had multiple effect sizes calculated from their data. All effect sizes and other relevant data were recorded in the *Meta-Essentials* workbook and an overall effect size was calculated using the random effects model. This overall effect size was used to indicate the degree of impact of TBI on visuospatial attention allocation. Subgroup analyses were conducted using the same workbook to determine the influence of different task designs and TBI factors, such as severity, on outcome. The impact of participant age and post-injury period were assessed using moderator meta-regression analyses in the combined effect size data and in subgroups.

A one-way analysis of variance (ANOVA) was conducted to determine whether the contribution of task design and severity were significantly different across subgroups. A two-way ANOVA was performed to determine how these effects interacted across TBI severity and task design. Subgroup analyses were also conducted to determine whether the type of outcome measures reported were significantly different, i.e., if the different behaviors were impacted differently by TBI.

## Results

**Figure 1:**
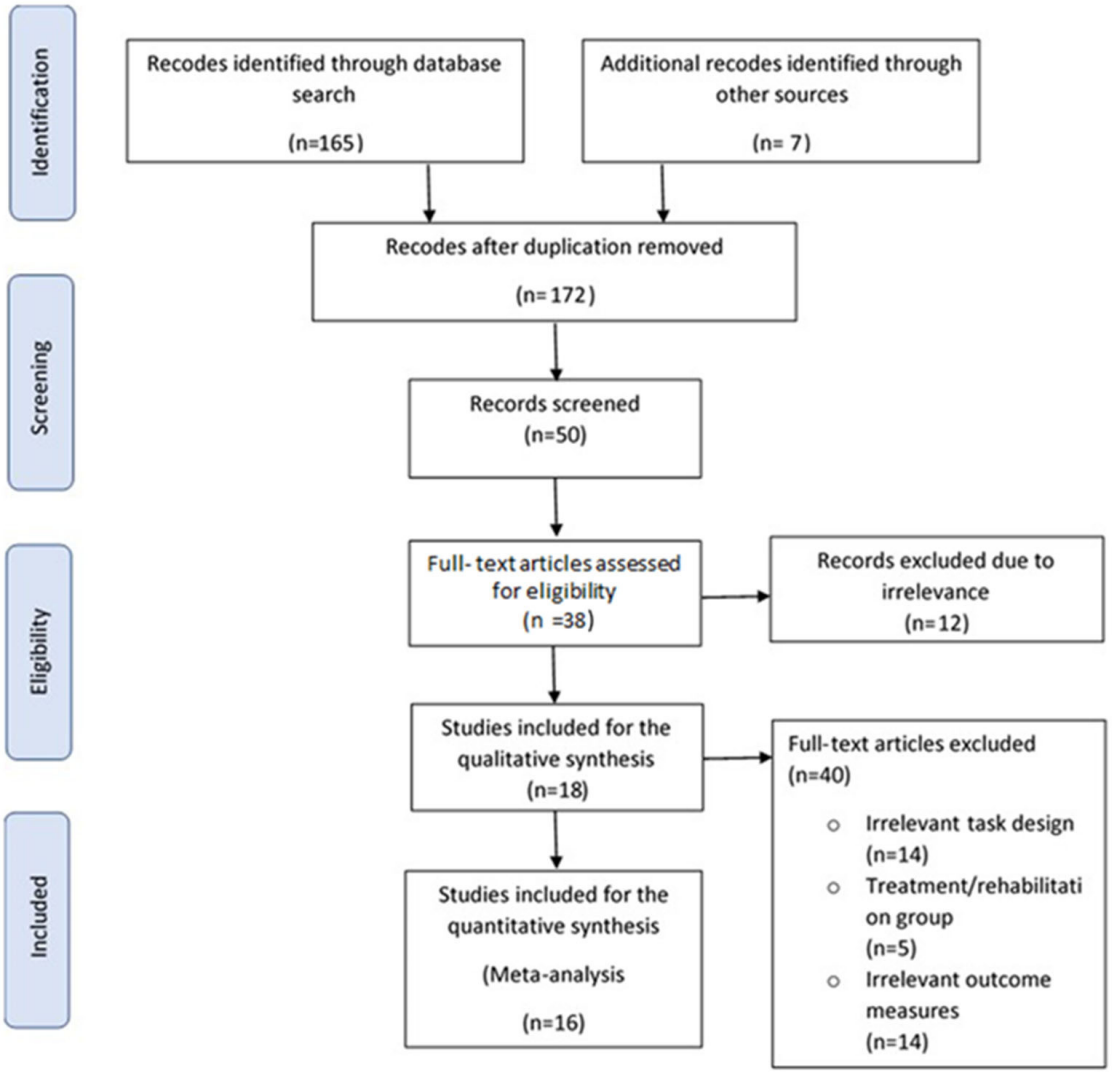
Flow diagram of study selection process.

The flow diagram in Figure 1 describes the study search and selection process and outcomes (Moher et al., 2009). Out of 50 abstracts screened, 18 studies met the inclusion criteria for qualitative analysis (Macflynn et al., 1984; Cremona-Meteyard et al., 1992; Cremona-Meteyard and Geffen, 1994b; Geldmacher and Hills, 1997; Hills and Geldmacher, 1998; Bate et al., 2001; Van Donkelaar et al., 2005; Halterman et al., 2006; Pavlovskaya et al., 2007; Catena et al., 2009; Kim et al., 2009; Sinnett et al., 2011; Rodríguez-Bailón et al., 2012; Hill-Jarrett et al., 2015; Schmitter-Edgecombe and Robertson, 2015; Robertson and Schmitter-Edgecombe, 2017; Shah et al., 2017; Hromas et al., 2020). After thorough review of the full texts, 16 of these 18 studies met the criteria for quantitative analysis, producing 80 calculated effect size estimates for meta-analysis. These included 359 TBI patients and 358 matched controls. The two studies (Kim et al., 2009; Hromas et al., 2020) from the qualitative analysis that were excluded from the meta-analysis were those that pooled data across task conditions preventing independent analysis of the type of attention processing. Studies were otherwise excluded from the review if they were case reports, case series, studies that had irrelevant task design, outcome measures and/or with intervention. Those studies that could not be accessed or for unextractable data where results were reported as median and range instead of mean and standard deviation/error.

### Qualitative Analysis

Eighteen studies were included in the systematic review. See Table 4 for summary of the qualitative analysis of these studies. Studies were assessed in regard to study quality, TBI definitions and criteria, recruitment methods, sample size, description of study participants including injury etiology, severity and post-injury period including follow-up visits, task design, and key outcome measures.

**Table 4:** Qualitative analysis.

**First author**	**Year**	**Severity**	**Subjects**	**Mean age (years)**	**Mean PIP (days)**	**TBI etiology**	**TBI diagnostic criteria**	**Task type**	**Outcome measures**
MacFlynn et al.	1984	Mild	45 Control 45 TBI	30.9	1 Follow-up 42 and 180	Not reported	Period of post-traumatic amnesia diagnosed by hospital staff	Visual search	Response time
Cremona-Meteyard et al.	1992	Moderate-to-severe	9 Control 11 TBI	29.3 (control) 30.1 (TBI)	2,176	Motor vehicle accident	Period of post-traumatic amnesia (>24 h) indicated by medical records	Covert orienting of attention task	Response time
Cremona-Meteyard et al.	1994	Mild	12 Control 9 TBI	22.1 (control) 23 (TBI)	14 Follow up 365 and 730	Sports-related injury	Period of loss of consciousness (2–20 min) and post-traumatic amnesia (<24 h) diagnosed by team coach and medical officers	Covert orienting of attention task	Response time
Geldmacher et al.	1997	Severe	21 Control 20 TBI	31.6 (control) 31.4 (TBI)	92.5	Not reported	Glasgow coma scale (3–8) indicated by medical records	Visual search	Q Score (accuracy and response time)
Hills et al.	1998	Severe	21 Control 20 TBI	31.6 (control) 31.4 (TBI)	92.5	Not reported	Glasgow coma scale (3–8) indicated by medical records	Visual search	Q Score (accuracy and response time)
Bate et al.	2001	Severe	35 Control 35 TBI	30.2 (control)28.9 (TBI)	843.8	Not reported	glasgow coma scale (3–8) and period of post-traumatic amnesia (>24 h) indicated by medical records	Covert orienting of attention task	Response time
Van Donkelaar et al.	2005	Mild	20 Control 20 TBI	21 (control)21 (TBI)	1.542	Mixed: sports and falls	American academy of neurology g2: period of disorientation (>15 min) and no loss of consciousness diagnosed by certified trainers	Attention network test	Response time
Halterman et al.	2006	Mild	20 Control 20 TBI	21 (control)21 (TBI)	1.542 Follow up 7, 14 and 28	Mixed: sports and falls	American academy of neurology g2: period of disorientation (>15 min) and no loss of consciousness diagnosed by certified trainers	Attention network test	Response time
Pavlovskaya et al.	2007	Severe	9 Control 21 TBI	23–47 (control) 18–47 (TBI)	90	Not reported	Glasgow coma scale (3–8) and period of loss of consciousness (>3d) diagnosed by hospital staff	Identification task with exogenous cue	Accuracy
Catena et al.	2009	Mild	20 Control 17 TBI	21 (control) 21 (TBI)	1.583 Follow up 6, 14, and 28	Mixed: falls, sports and collisions	American academy of neurology g2: period of disorientation (>15 min) and no loss of consciousness. diagnosed by certified trainers	Attention network test	Response time
Kim et al.	2009	Moderate	15 Control 17 TBI	25.1 (control) 27.8 (TBI)	480	Not reported	Glasgow coma scale (9–12) indicated by medical records	Covert orienting of attention task	Response time
Sinnett et al.	2011	Mild	10 Control 8 TBI	22 (control) 35 (TBI)	80.1	Not reported	Not reported	Temporal order judgement covert orienting of attention task (with endogenous and exogenous cues)	Point of subjective simultaneity
Rodríguez-Bailón et al.	2012	Severe	9 Control 9 TBI	30.89 (control) 29.4 (TBI)	Not reported	Not reported	Lesion on neuroimaging indicated by medical records	Attention network test	Response time
Hill-Jarrett et al.	2015	Moderate-to-severe	12 Control 12 TBI	25.1 (control) 28.7 (TBI)	2,091	Not reported	Glasgow coma scale, period of post-traumatic amnesia and loss of consciousness indicated by medical records	Attention network test	Response time
Schmitter-Edgecombe et al.	2015	Moderate-to-severe	40 Control 40 TBI	28.83 (control) 31.43 (TBI)	41.2 Follow up 305	Mixed: motor vehicle accidents, sports, falls, assault	Glasgow coma scale (<12) and period of post-traumatic amnesia diagnosed by paramedics or hospital staff	Visual search	Response time
Robertson et al.	2017	Moderate-to-severe	30 Control 30 TBI	29.87 (control) 30.43 (TBI)	38.7	Mixed: motor vehicle accidents, sports, falls, assault	Glasgow coma scale (<12) and period of post-traumatic amnesia diagnosed by paramedics or hospital staff	Visual search and visual search with endogenous cue	Response Time
Shah et al.	2017	Mixed	24 Control 13 TBI	43 (control) 45 (TBI)	745.38	Not reported	American congress of rehabilitation medicines guidelines: loss of consciousness indicated by medical records	Attention network test	Response time
Hromas et al.	2020	Moderate-to-sever	12 Control 12 TBI	24.8 (control) 28.7 (TBI)	1,791	Mixed: motor vehicle accidents, bicycle accident, animal accident, falls	Glasgow coma scale, period of post-traumatic amnesia and loss of consciousness indicated by medical records	Attention network test	Response time

*PIP, Post-injury period*.

### Study Quality, TBI Definitions, and Criteria

All studies were well-designed case-control studies, as per the inclusion criteria, and included a thorough description of the aims, definitions, and procedures. The diagnostic criteria used for classifying TBI varied between studies, but the majority, ~89%, indicated they utilized a measure of loss of consciousness, post-traumatic amnesia, alteration of mental state, Glasgow Coma Scale, or a combination thereof. Two studies (Macflynn et al., 1984; Cremona-Meteyard et al., 1992) classified injury severity using period of post-traumatic amnesia alone, while GCS score was used as the sole indicator in three studies (Geldmacher and Hills, 1997; Hills and Geldmacher, 1998; Kim et al., 2009). The duration of loss of consciousness was used alone in one study (Shah et al., 2017), in accordance with the American Congress of Rehabilitation Medicines Guidelines (Ruff et al., 2009). American Academy of Neurology grading system (American Academy of Neurology, 1997), which refers to both an alteration of mental state and the period of loss of consciousness, was used in three studies (Van Donkelaar et al., 2005; Halterman et al., 2006; Catena et al., 2009). Another combination of at least two factors from period of loss of consciousness, duration of post-traumatic amnesia or GCS score were used in seven studies (Cremona-Meteyard and Geffen, 1994b; Bate et al., 2001; Pavlovskaya et al., 2007; Hill-Jarrett et al., 2015; Schmitter-Edgecombe and Robertson, 2015; Robertson and Schmitter-Edgecombe, 2017; Hromas et al., 2020). One study (Sinnett et al., 2011) did not report their diagnostic criteria, and one study (Rodríguez-Bailón et al., 2012) used neuroimaging as their primary criterion.

Nine of the 18 included studies used medical records to confirm diagnosis of TBI (Cremona-Meteyard et al., 1992; Geldmacher and Hills, 1997; Hills and Geldmacher, 1998; Bate et al., 2001; Kim et al., 2009; Rodríguez-Bailón et al., 2012; Hill-Jarrett et al., 2015; Shah et al., 2017; Hromas et al., 2020). Diagnosis by hospital casualty staff or paramedics was required for four studies (Macflynn et al., 1984; Pavlovskaya et al., 2007; Schmitter-Edgecombe and Robertson, 2015; Robertson and Schmitter-Edgecombe, 2017), and diagnosis by a trained medic, coach or team trainer was required for four studies (Cremona-Meteyard and Geffen, 1994b; Van Donkelaar et al., 2005; Halterman et al., 2006; Catena et al., 2009). One study (Sinnett et al., 2011) did not report the diagnosis method.

### Methodology, and Outcome Measures

The task designs and methodology were well-described amongst all the studies, as per the inclusion criteria. The task designs involved various visual search tasks, tasks with a central directional or endogenous cue, and tasks with a peripheral spatial or exogenous cue. There were four studies (Macflynn et al., 1984; Geldmacher and Hills, 1997; Hills and Geldmacher, 1998; Schmitter-Edgecombe and Robertson, 2015) that utilized only visual search tasks without cueing. One study (Kim et al., 2009) involved task conditions that used only an endogenous cue and one study (Pavlovskaya et al., 2007) used only exogenously cued conditions. The rest of the studies used more than one condition; four studies (Cremona-Meteyard et al., 1992; Cremona-Meteyard and Geffen, 1994b; Bate et al., 2001; Robertson and Schmitter-Edgecombe, 2017) used both endogenously cued and un-cued condition, seven studies (Van Donkelaar et al., 2005; Halterman et al., 2006; Catena et al., 2009; Rodríguez-Bailón et al., 2012; Hill-Jarrett et al., 2015; Shah et al., 2017; Hromas et al., 2020) used both an un-cued condition and an exogenously cued task, and one study (Sinnett et al., 2011) used both cued task types. Of the 14 studies that used some type of cued condition, six referred to the Covert Orienting of Attention Task (Posner et al., 1980; Posner and Cohen, 1984), and seven referred to the Attention Network Test (Fan et al., 2005) as the basis for task design.

Studies typically reported either response time or accuracy as their key outcome measures. Fifteen studies either reported response time or comparative measure of response time between tasks (Macflynn et al., 1984; Cremona-Meteyard et al., 1992; Cremona-Meteyard and Geffen, 1994b; Bate et al., 2001; Van Donkelaar et al., 2005; Halterman et al., 2006; Catena et al., 2009; Kim et al., 2009; Sinnett et al., 2011; Rodríguez-Bailón et al., 2012; Hill-Jarrett et al., 2015; Schmitter-Edgecombe and Robertson, 2015; Robertson and Schmitter-Edgecombe, 2017; Shah et al., 2017; Hromas et al., 2020). One study (Pavlovskaya et al., 2007) reported only the fraction correct as the key outcome and two studies (Geldmacher and Hills, 1997; Hills and Geldmacher, 1998) reported a “Q score” which combined measures of accuracy and response time.

### Study Participants

The recruitment processes of the studies were well-documented. Three studies (Cremona-Meteyard et al., 1992; Kim et al., 2009; Rodríguez-Bailón et al., 2012) did not specifically mention their recruitment source. Nine studies (Macflynn et al., 1984; Geldmacher and Hills, 1997; Hills and Geldmacher, 1998; Bate et al., 2001; Pavlovskaya et al., 2007; Schmitter-Edgecombe and Robertson, 2015; Robertson and Schmitter-Edgecombe, 2017; Shah et al., 2017; Hromas et al., 2020) recruited directly from hospitals, rehabilitation clinics, or out-patient services. Two studies (Sinnett et al., 2011; Hill-Jarrett et al., 2015) advertised publicly or within university groups and four studies (Cremona-Meteyard and Geffen, 1994b; Van Donkelaar et al., 2005; Halterman et al., 2006; Catena et al., 2009) recruited from sports teams or athletics programs.

From these groups, studies were characterized as having a *selected* or *unselected* recruitment process (Belanger et al., 2005). Those studies that recruited from rehabilitation centers or hospital out-patient services were classified as selected because recruiters selected patients who were referred to these services for specific symptom management following injury. Studies that did not mention their recruitment process or recruited from sports programs, public advertisement, or hospital emergency departments were classified as unselected as patients were not recruited based on presentation of specific symptoms. Based on this classification, eight studies (Geldmacher and Hills, 1997; Hills and Geldmacher, 1998; Bate et al., 2001; Pavlovskaya et al., 2007; Schmitter-Edgecombe and Robertson, 2015; Robertson and Schmitter-Edgecombe, 2017; Shah et al., 2017; Hromas et al., 2020) had a selected recruitment process and 10 studies (Macflynn et al., 1984; Cremona-Meteyard et al., 1992; Cremona-Meteyard and Geffen, 1994b; Van Donkelaar et al., 2005; Halterman et al., 2006; Catena et al., 2009; Kim et al., 2009; Sinnett et al., 2011; Rodríguez-Bailón et al., 2012; Hill-Jarrett et al., 2015) had an unselected process. No quantitative analysis of these groups was conducted due to bias in injury severity groups. All the studies with a selected recruitment process were of moderate-to-severe or severe TBI, while the majority (66.67%) of unselected studies were of mild TBI.

The average age of TBI patients was 29.1 years, and that of the matched controls was 27.9 years. Etiology of TBI from one study (Cremona-Meteyard et al., 1992) was motor-vehicle accidents, while one other reported only sports-related concussions (Cremona-Meteyard and Geffen, 1994b). All other studies either did not report etiology or reported mixed etiology among participants.

### Quantitative Analysis (Meta-Analysis)

Of the studies included in this review, 16 studies presented sufficient data for meta-analysis. Studies reported measures of either accuracy or response time and effect size estimates were generated as comparisons of performance in their respective tasks. In order to ensure that these different outcome measures did not significantly affect the analysis, further investigation was conducted (see subgroup analysis below). The majority of studies reported multiple comparisons across task conditions and different time points. As a result, multiple effect sizes were calculated for most studies, one for each appropriate task condition or post-injury period, at an average of five effect sizes per study. In total, 80 effect size estimates of visuospatial attention were calculated.

### Overall Effect Size and Heterogeneity

In the meta-analysis, in addition to the overall effect size, the effect sizes for different TBI severity groups and task conditions were also considered for subgroup analyses. The reporting of mixed etiologies, or lack of reported etiology (see qualitative analysis) by the majority of studies prevented comparison of different etiology groups. Meta-regression analysis was conducted to determine the change in effect size as a result of post-injury period. This investigation would help determine whether this attention deficit is chronic or recovers with time. An additional meta-regression analysis was conducted to determine whether and how the impact of TBI is dependent on age.

**Figure 2:**
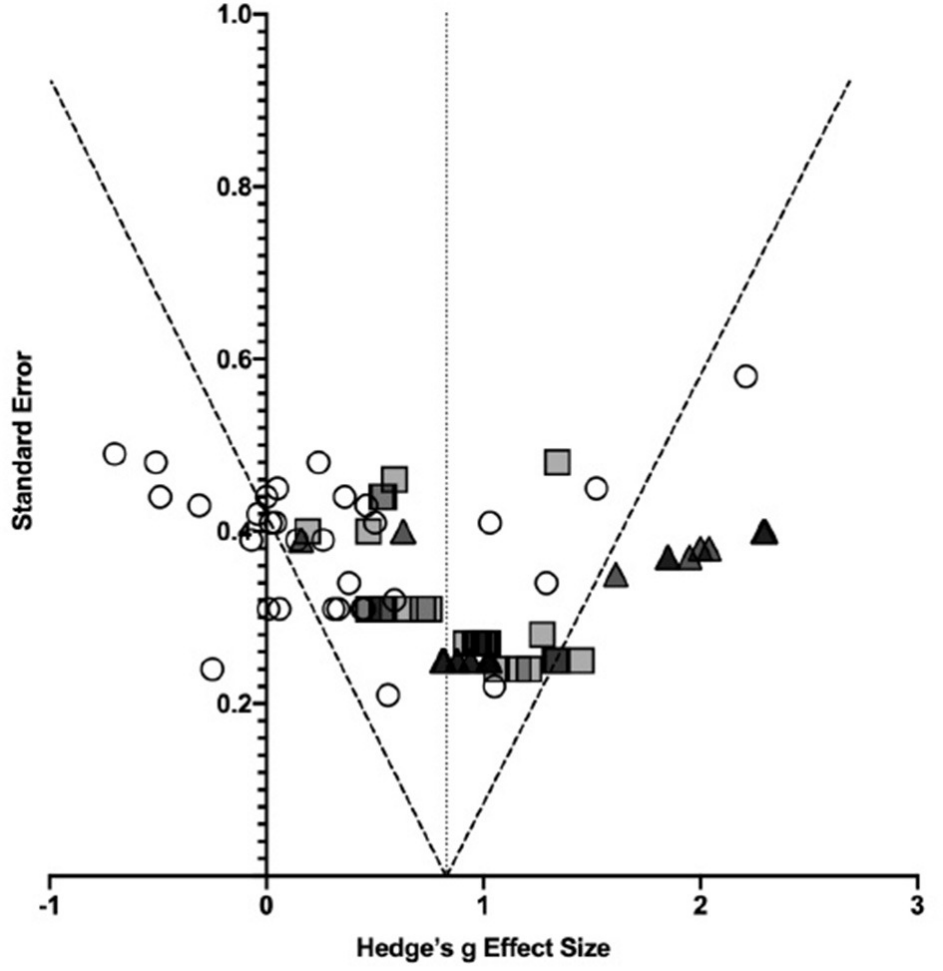
Effect sizes plotted as a function of standard error. Symbols represent individual effect size estimates. Circles represent effect size estimates for mild TBI, squares represent moderate-to-severe TBI effect sizes, and triangles represent severe TBI estimates. Dotted lines represent 95% confidence intervals.

In order to provide a visual representation of publication bias, a funnel plot (Figure 2) was used. There was a broad range of effect sizes observed and when assessed using an Egger regression statistical analysis, there was significant publication bias observed (*p* = 0.035). Figure 2 shows the 80 effect size estimates plotted against standard error. The different symbols represent the severity subgroups explored below. From the figure it can be inferred that this statistical evidence of publication bias is likely driven by study differences particularly trends in injury severity.

From the 80 effect size estimates, the overall estimated effect size of TBI on visuospatial attention was 0.79 (SE: 0.07, 95% CI: ±0.14, *I*^2^ = 68.39%). Figure is a forest plot including individual and combined effect sizes. This large, combined effect size was significantly different from zero (*Z* = 11.42, *p* < 0.0001) with medium to high heterogeneity which highlights the potential role of study design and methodology, injury severity, post-injury period and outcome measures as contributing factors. Though heterogeneity was observed, these results indicate that visuospatial attention is significantly and largely affected by TBI at all severities and across all attention allocation types however the variation between individual effect sizes suggests the nature of this effect is likely to differ within these domains.

Further investigation of the causes of this degree of heterogeneity was warranted. Therefore, additional subgroup and moderator analyses were conducted to determine whether and how this deficit in visuospatial attention following TBI was affected by severity of TBI, time since injury, and participants age, as well as further analyses on task design, type of attention allocation and outcome measure.

### Subgroup Analyses: Effect of Severity, Task Type, and Outcome Measure

**Figure 3:**
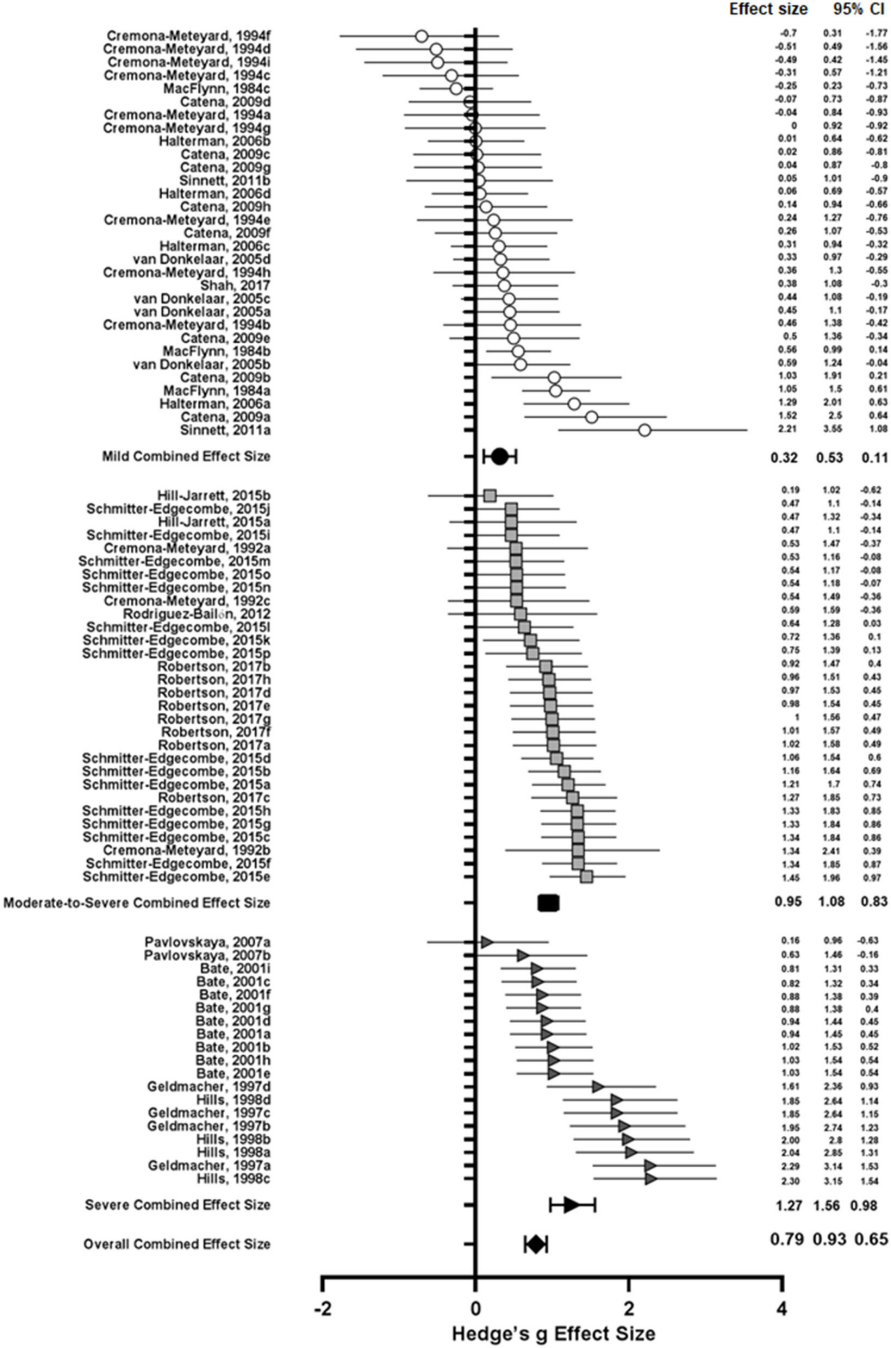
Open and shaded symbols represent individual effect sizes while closed symbols represent combined effect sizes. Circles represent effect sizes from studies of mild TBI, squares represent moderate-to-severe TBI studies, and triangles represent severe TBI studies. Error bars represent 95% confidence intervals.

Subgroup analyses of injury severity produced three effect sizes (see Table 5). As reported in the qualitative analysis, the literature typically grouped patients as mild (*N* = 30), moderate-to-severe (*N* = 30), or severe (*N* = 19), hence the effect size estimates were grouped in the same way. The results of this analysis are shown in Figure for different severity levels. The combined effect size for mild TBI was 0.32 with medium heterogeneity (*I*^2^ = 55.81%, *Q* = 65.62), while that of moderate-to-severe TBI was 0.95 with low heterogeneity (*I*^2^ = 23.08%, *Q* = 37.70), and for severe TBI the effect size estimate was 1.27 (*I*^2^ = 68.43%, *Q* = 57.02). A one-way between groups ANOVA comparing the effect of injury severity on visuospatial attention following TBI showed a significant between different subgroups [*F*_(2, 76)_ = 24.14, *p* < 0.0001]. *Post-hoc* analysis using Tukey's multiple comparisons test revealed that the combined effect size estimate for mild TBI was significantly different from and lower than the estimates for both moderate-to-severe [mean difference (*MD*) = −0.63, *p* < 0.0001] and severe TBI (*MD* = −0.95, *p* < 0.0001), however moderate-to-severe TBI was not significantly different from severe TBI (*p* = 0.0759) (see Figure 3). The lack of significant difference between these groups may be explained by the literature grouping. Particularly, if the data could be separated into “moderate” and “severe” groups.

**Table 5:** Subgroup analysis of severity by task condition including effect size estimates and heterogeneity.

	**Mild subgroup**	**Moderate-to-severe subgroup**	**Severe subgroup**
Visual search task	0.32	0.98	1.64
	*I*^2^ = 63.89% *Q* = 22.16 *N* = 9	*I*^2^ = 30.12% *Q* = 28.62 *N* = 21	*I*^2^ = 68.30% *Q* = 31.55 *N* = 11
Endogenous attention task	−0.04	1.02	0.93
	*I*^2^ = 0% *Q* = 5.83 *N* = 7	*I*^2^ = 0% *Q* = 2.58 *N* = 6	*I*^2^ = 0% *Q* = 0.90 *N* = 6
Exogenous attention task	0.48	–	–
	*I*^2^ = 58.70% *Q* = 31.47 *N* = 14	*N* = 3 N/A N/A	*N* = 2 N/A N/A

Subgroup analysis by task condition was also conducted. Initially, effect sizes were separated into visual search tasks, endogenous attention tasks, and exogenous attention tasks. The combined effect sizes for these groups were 0.98 (*I*^2^ = 72.42%, *Q* = 145.01), 0.70 (*I*^2^ = 53.22%, *Q* = 38.48), and 0.45 (*I*^2^ = 40.57%, *Q* = 31.97) respectively. Importantly, this indicates that TBI patients perform worse in higher order, attention demanding tasks such as visual search and endogenous attention allocation tasks compared with the bottom-up attention processing involved in exogenous attention tasks. As before, a one-way ANOVA was conducted to determine the effect of task type on attention processing following TBI. There was a significant effect of task type on performance by TBI patients [*F*_(2, 77)_ = 5.660, *p* = 0.0051). Tukey's multiple comparisons *post-hoc* test was conducted to further investigate these relationships. Visual search task performance was significantly different from exogenous attention task performance (*MD* = 0.53, *p* = 0.0042), however performance in endogenous attention tasks was not significantly different from either of the other conditions (*p* > 0.2).

In order to further investigate the heterogeneity evident in these subgroups, a further breakdown of groups by task type was performed. The subgroups involved visual search tasks (*N* = 31), no cue conditions of endogenous and exogenous attention tasks (*N* = 10) (previously included in the visual search group), endogenous task with valid cue (*N* = 9), endogenous task with invalid cue (*N* = 10), and exogenous task (*N* = 20). The lack of available data for the exogenous invalid cue condition prevented meaningful analysis on cue type in this task. The combined effect size estimates for these subgroups respectively, were 1.13 (*I*^2^ = 73.49%, *Q* = 113.16), 0.54 (*I*^2^ = 39.71%, *Q* = 14.93), 0.92 (*I*^2^ = 0%, *Q* = 6.18), 0.49 (*I*^2^ = 69.12%, *Q* = 29.14), and 0.45 (*I*^2^ = 40.57%, *Q* = 31.97). A one-way ANOVA was conducted to determine the relationship between task design and reported visuospatial attention deficit. A significant relationship was identified [*F*_(4, 75)_ = 6.159, *p* = 0.0002]. Visual search performance was significantly different from the no cue condition (*MD* = 0.59, *p* = 0.0428), endogenous invalid cue condition (*MD* = 0.64, *p* = 0.0214), and exogenous task condition (*MD* = 0.68, *p* = 0.0005). No other task comparisons were statistically significant (*ps* > 0.15). Clearly, despite this detailed analysis there is still a great deal of heterogeneity involved in some of the groups. This implicates injury severity, time since injury, and age as possible factors as changes in task design have not fully explained the variability.

An alternative investigation into the heterogeneity among task conditions is by TBI severity. Seven effect size estimates were calculated and are listed in their appropriate subgroups in Table 5.

Effect size estimates were not calculated for exogenous attention tasks in moderate-to-severe or severe TBI as these groups contained only three and two effect sizes, respectively and therefore insufficient for analysis. Qualitatively, the results reported for these groups were systematically smaller than the other subgroups for the same degree of injury severity. Additionally, the heterogeneity measures (*I*^2^) for endogenous attention tasks were 0% for all severity groups suggesting that these groups may also be over-analyzed. The results, therefore, should be interpreted with caution.

Further analysis of task condition in severity groups was not conducted so as to avoid over-analyzing data. Some conditions had only 1 or 2 effect sizes involved in their calculation when groups were broken down to the cue validity level, which would over-represent these effects. Qualitatively, however, when further investigation was conducted into the negative effect size produced in the endogenous attention task for mild TBI condition, all the negative effect sizes were attributed to invalid cueing conditions. These negative effect sizes suggest better performance by TBI patients than controls in this task condition. This lends weight to the supposition that TBI patients may not properly allocate attention with the directional cue. Hence, they do not exhibit an increase in reaction time when forced to reorient to a different target location, but rather treat the task more akin to a simple visual search condition.

The majority of studies reported response or completion time data, however, few studies reported data indicating task accuracy. A subgroup analysis of these data types was performed to determine if there were any differences in combined effect size and heterogeneity between these groups. The combined effect size for the subgroup that reported accuracy data was 1.67 (*I*^2^ = 69.60%, *Q* = 29.60) while the subgroup for response time data was 0.69 (*I*^2^ = 58.58%, *Q* = 166.58). These results indicate that whilst these groups have a similar degree of variability, accuracy is affected to a much greater degree than response time. However, this result may be confounded by the fact that the only studies that reported accuracy data were those of severe TBI patients, exacerbating the effect, and warrants further investigation.

### Meta-Regression Analysis: Effect of Post-injury Period and Age

Preliminary analysis of the relationships between effect size, post-injury period, and age indicated signs of Simpson's Paradox in severity groups due to variance in sampling over participant age and post-injury period (Blyth, 1972; Wagner, 1982). In order to account for this, meta-regression analyses were conducted for each severity subgroup explored above. In both analyses, effect sizes were weighted by sample size.

**Figure 4:**
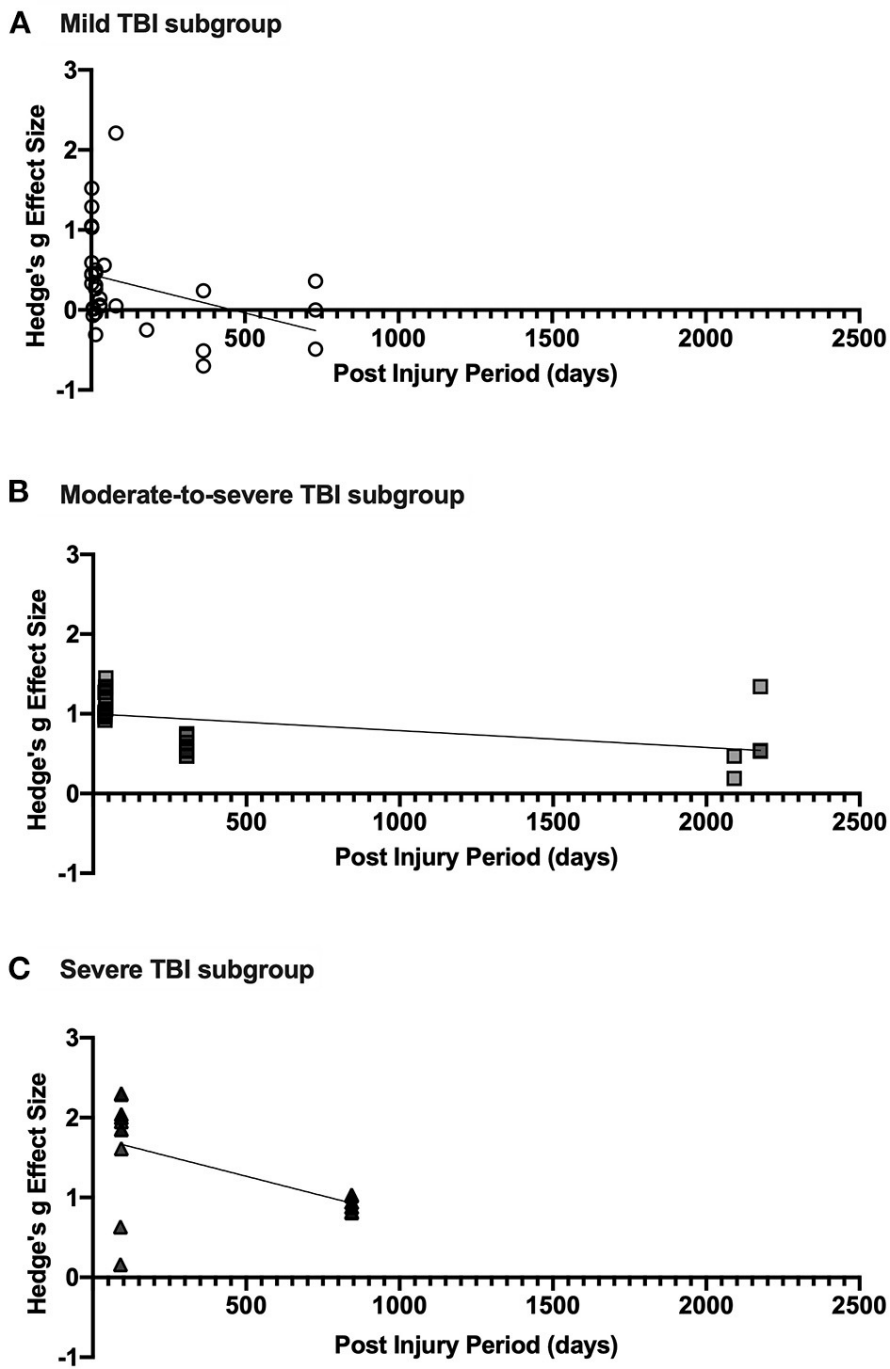
**(A)** shows effect size estimates for studies of mild TBI, **(B)** shows that of moderate-to-severe TBI, and **(C)** includes effect sizes for severe TBI. Each figure includes the linear regression line estimate for each group.

The results of the post-injury period meta-regression analyses are presented in Figure 4. For mild TBI, the relationship between post-injury period and effect size was statistically significant (*p* = 0.031). The regression line shown in Figure 4A intercepts the X-axis at ~460 days, indicating recovery of visuospatial attention deficit over time. Similarly, the trend in moderate-to-severe TBI was also statistically significant (*p* = 0.002). Whilst the regression line does not cross the X-axis (see Figure 4B) in the sampled time period, the projected X-intercept occurs at 4,741 days, or ~13 years. In this analysis, the study by Rodríguez-Bailón et al. (2012) was excluded as they did not report time since injury. In severe TBI, the relationship was also statistically significant (*p* < 0.0001) with predicted X-intercept at 1,789 days (see Figure 4C). However, the lack of studies with varied post-injury periods is likely to have impacted this analysis and additional data may produce a different result.

**Figure 5:**
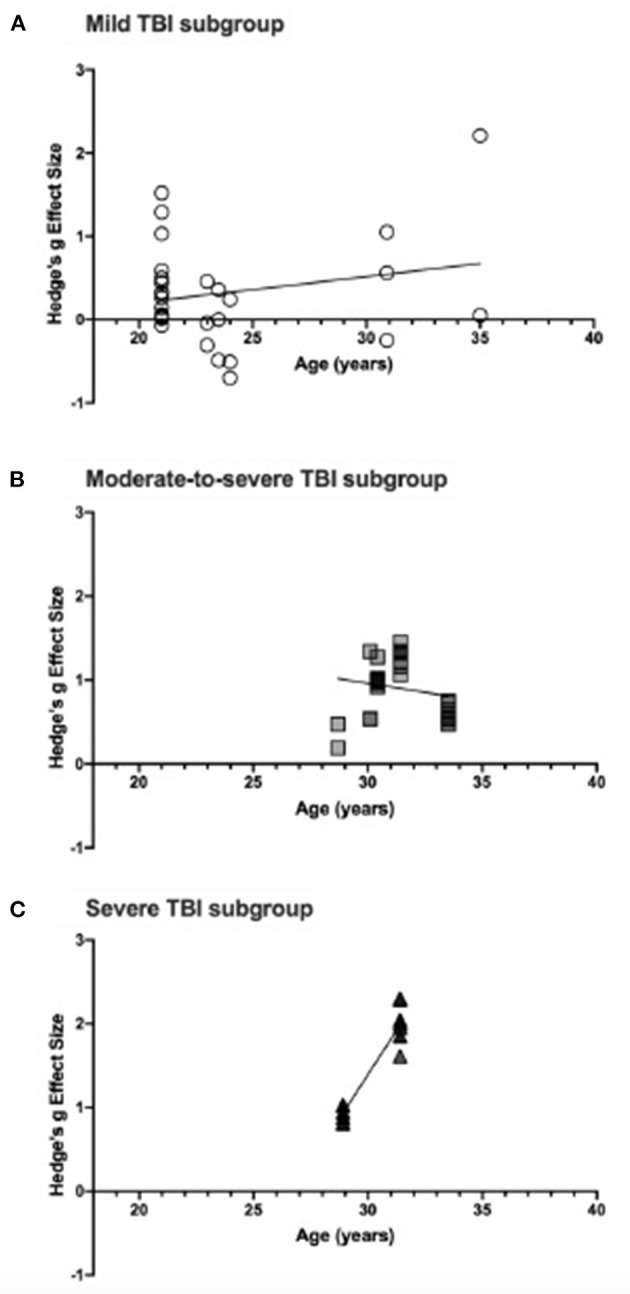
**(A)** shows effect size estimates for studies of mild TBI, **(B)** shows that of moderate-to-severe TBI, and **(C)** includes effect sizes for severe TBI. Each figure includes the linear regression line estimate for each group.

Meta-regression analyses of the relationship between participant age and effect size estimates were conducted in severity subgroups and the results are presented in Figure 5. In mild TBI, the relationship between effect size and age was not statistically significant (see Figure 5A), suggesting that age does not have a significant impact on attention deficit due to injury. The relationship for moderate-to-severe TBI, shown in Figure 5B, was not significant (*p* = 0.095) indicating no improvement of attention deficit with age. Importantly, however, the lack of varied sampling (age range: 28.7–33.52 years) may have impacted the analysis and data reporting a wider age range could impact the results. Similarly, the relationship in severe TBI was statistically significant (*p* < 0.0001) but lacked a variety of age samples (see Figure 5C). In the severe TBI subgroup, Pavlovskaya et al. (2007) was excluded from this meta-regression as they reported age range only.

## Discussion

The purpose of the present study was to investigate, both qualitatively and quantitatively, the current evidence of visuospatial attention deficit in adults with TBI. Qualitative synthesis of previous studies on the topic assessed discrepancies in task designs, recruitment processes, and severity groups. An overall effect size and heterogeneity was generated through meta-analysis of the available response time and accuracy data assessing visuospatial attention following TBI. Additionally, subgroup meta-regression analyses were performed that examined the contribution of injury severity, task design, post-injury period, and age as moderating factors. Although, the assessment of visuospatial attention deficits is not a mainstream approach in the assessment of cognitive function in TBI, the present study has shown that its potential utility as means of assessing whether and the extent to which different aspects of attention are affected by injury. This may lead to further research and end user benefits (in clinical practice and assessment) that provide more efficient and sensitive tests to detect and monitor deficits in executive function following TBI. Furthermore, in depth understanding of the effects of TBI on visual attention may in the future inform education and health policy regarding the impact of TBI on cognitive functioning and everyday behavior.

The qualitative analysis investigated visuospatial attention deficits following mild, moderate-to-severe, or severe TBI as reported by previous studies. The types of attention tasks were either visual search tasks or required endogenous or exogenous allocation of attention with a visual cue and the majority of reported outcome measures were response times. Quantitative assessment of the presented data, which included 80 calculated effect size estimates from 16 studies, revealed a significant and large deficit in visuospatial attention following TBI (overall effect size = 0.79), however, there was a moderate degree of heterogeneity across studies (*I*^2^ = 68.39%). A large effect size indicates impaired attention allocation as a result of TBI and lends support to the use of such tasks as measures for characterizing cognitive outcome after injury. These findings are unique as this is the first review of this type of attention processing in TBI, however, previous meta-analyses of neuropsychological outcome following injury do support the notion of attention deficit after injury (Binder et al., 1997; Zakzanis et al., 1999; Belanger et al., 2005; Frencham et al., 2005).

A key source of heterogeneity in this analysis was TBI severity, with a small effect reported by studies of mild TBI (*g* = 0.32) and large deficits reported by studies of moderate-to-severe (*g* = 0.95) and severe TBI (*g* = 1.27). The effect size for mild TBI was significantly different from both moderate-to-severe and severe TBI, indicating greater deficits in attention allocation capacity with increased injury severity, as supported by previous reviews of neuropsychological outcome and attention following TBI (Hoofien et al., 2001; Dan Hoofien et al., 2002; Ponsford et al., 2008). The moderate-to-severe effect size estimate was not significantly different from the severe subgroup effect size, however, the lack of distinction between moderate and severe grades of injury in the literature likely contributed to this relationship. The serial increase in effect size between these groups, whilst not statistically significant, would suggest that separating data into moderate and severe subgroups would reduce variance, producing a significant difference.

Task design was another major source of variability and the different task types produced significantly different effect sizes between tasks, and across severity groups. Particularly, visual search (*g* = 0.98) and endogenous attention allocation tasks (*g* = 0.70) produced larger deficits when compared to exogenous attention tasks (*g* = 0.45) indicating that higher order attention processing is affected to a greater degree than lower order systems. Such processing dysfunction is consistent with similar deficits in executive function (Rabinowitz and Levin, 2014) and oculomotor functions in TBI (Mani et al., 2018) Both executive function and oculomotor function are highly linked with attention processing (Posner and Cohen, 1984; Corbetta and Shulman, 2002; Hunt et al., 2019) so it stands to reason that the patterns seen in these functions, characterized by more severe high order deficits, would be emulated by the trends in attention processing reported here.

One area of interest was the effect of cue validity on response time following TBI. Whilst there was no significant effect of validity within the endogenous task, when compared with a baseline visual search condition a significant difference was identified with endogenous invalid condition. Importantly, the cued condition had a significantly smaller effect size than the visual search task. Given that the majority of effect sizes referred to response time, this would indicate that in the endogenous invalid condition, TBI patients are less impaired than in the visual search condition. Despite an overall slowed cognitive processing, this would suggest relatively intact spatial reorienting ability. On the other hand, the endogenous valid cue condition was not significantly different from the visual search condition, indicating impaired attention allocation with a directional cue. Collectively, these results indicate impaired endogenous attention allocation. The reduced effect size in the invalid cue condition may be linked to a lack of attention allocation with the cue. By failing to properly utilize the cue, TBI would appear to treat the task more akin to a visual search task and do not need to reorient attention thus producing reduced response time cost when compared to controls. This is supported by the qualitative evidence that in the mild TBI subgroup, all but one effect size for the endogenous invalid condition was negative suggesting better performance in this task by the TBI groups.

In the exogenous task, a significant relationship was identified with the visual search condition. The significantly smaller effect size in the cued task would indicate relatively intact bottom-up attention allocation. It is important to note that only 2 of the included studies involved an invalid exogenous cue condition hence there was no meaningful analysis able to be conducted for this condition. There is much need for research targeted toward clarifying the degree of deficit in exogenous allocation of attention, particularly including an invalid condition.

The particular nature and extent of these attention deficits following TBI are unclear. Future research should aim at isolating and characterizing their implications in the lives of patients. Some potential areas of interest are endogenous attention influences on binocular rivalry and bistable perception. The former was addressed in healthy individuals by Chong et al. (2005) and the latter investigated by Brouwer and van Ee (2006). Chong and colleagues identified prolonged dominance durations in binocular rivalry tasks in healthy individuals when endogenous attention is engaged. They also simulated the effect of this attention engagement by increasing the contrast of the dominant stimulus, suggesting that top-down attention control engages a bottom-up perceptual change to increase salience and maintain attention engagement. Since endogenous attention engagement appears to be impaired in TBI, the same effect may not be identified in injured participants. Further, the impact of engaging exogenous attention by increasing stimulus salience may be lost, reduced or even exacerbated following TBI. Exacerbation of the effect could indicate difficulty with disengaging exogenous attention, an area which has been sparsely investigated to date as identified by the present study.

Brouwer and van Ee (2006) found that the physical parameters of a perceptually bistable stimuli constrain endogenous attention control mechanisms in controls. The voluntary engagement of top-down attention control decreased stability durations overall, however the degree of change was dependent on changes in the exogenous stimulus characteristics, dot-density, and angular velocity. Here, again, it is the relationship between endogenous and exogenous attention that is critical to high order perception. The identified attentional deficits following TBI may see a reduced impact of voluntary perceptual switching as endogenous attention engagement is impaired and therefore unable to help actively switch between the two possible interpretations of the ambiguous stimulus. In addition, impairment in global dot form and motion perception (Alnawmasi et al., 2019) after injury may affect perceptual stability. This is particularly relevant as Brouwer and van Ee indicated that “competition between perceptual interpretations during structure-from-motion appears to occur between surface-based representations rather than between individual elements” (Brouwer and van Ee, 2006, p. 3393). Impaired global dot motion perception may alter or impede perception of a bistable structure-from-motion stimulus in individuals who have sustained TBI. Compounding this, if bistable perception is successful, TBI patients may exhibit reduced ability to voluntarily switch between interpretations by engaging endogenous attention and hence rely on exogenous stimulus characteristics to drive perception. Since higher order processing is significantly affected as a consequence of TBI, conducting these kinds of attention dependent high order tasks in a TBI population may help illustrate the nature of deficit and aid in the development of more optimal therapeutics and rehabilitation programs for patient care.

Subgroup analysis of the reported outcome measure revealed significantly larger effect sizes from measures of accuracy as compared to response time. This would implicate impaired decision-making rather than slowed processing as the key issue following TBI. Whilst slowed cognition is a well-researched phenomenon associated with TBI (Madigan et al., 2000; O'Jile et al., 2006; Willmott et al., 2009; Dymowski et al., 2015) impaired decision-making has also been identified following injury, particularly moderate and severe injuries (Martens et al., 2012, 2013; Wood and McHugh, 2013). In light of this, it is likely that both factors contribute to the effects reported in the present study. It should also be noted that the accuracy subgroup consists of studies of severe TBI only, hence the reported effect may have been artificially inflated by these larger effect sizes.

Meta-regression analyses within severity subgroups revealed significant improvement in visuospatial attention over time following injury. All severity groups showed statistically significant improvement with post-injury period, although only the mild TBI group suggested complete recovery within the sampled time period. These regression analyses lend weight to the possibility for these task measures to be used in the monitoring and prognostication of TBI. They may also be used to help determine the efficacy of treatment and rehabilitation programs in improving cognitive function after injury.

There is significant clinical need for reliable, accurate and cost-effective markers of attention deficit following injury (Dambinova et al., 2016; Wang et al., 2018) and these tasks, if designed systematically to reduce variability, may be able to help satisfy this need. While neuroimaging techniques are often used as primary indicators for cognitive function (Levin et al., 1987; Eisenberg and Levin, 1989; Belanger et al., 2007; Mayer et al., 2011; Rabinowitz and Levin, 2014; Dambinova et al., 2016) these are impractical and too expensive to be used in regular clinical practice and condition management. Hence, investigation into other technologies that may be used in conjunction with these tasks to assist in determining the level of cognitive deficit after injury is a growing area of research. In particular, video-based eye-tracking technology for eye movement and pupil size monitoring has become an area of increasing interest (Ciuffreda et al., 2017; Gallaway et al., 2017; Capó-Aponte et al., 2018; Mani et al., 2018; Walz et al., 2020). There is immense potential in this area as pupillometry and eye-tracking provide fast, non-invasive and objective measures of attention processing (Daniels et al., 2012; Hunt et al., 2019; Lasaponara et al., 2019) and have been used in the past to identify and quantify attention deficit in TBI (Heitger et al., 2009; Hunt et al., 2016; Snegireva et al., 2018; Walz et al., 2020) and other cognitive and neurodegenerative disorders (Karatekin et al., 2010; MacAskill and Anderson, 2016; Wang et al., 2016; Granholm et al., 2017; Turi et al., 2018).

When meta-regression analyses were conducted in severity subgroups, there was no significant change in effect size with participant age, except for the severe TBI group which had very limited age range sampling. There is a need for more data in order to say this with certainty, but the evidence at this stage indicates that the impact of TBI on visual attention is the same across all age groups.

TBI causes a large degree of visuospatial attention deficit. The degree of deficit increases with injury severity although it does show improvement over time at all severity levels. Importantly, high order attention processes such as endogenous allocation of attention and the complex processes involved in un-cued visual search are affected to a greater degree than lower order, bottom-up attention processes. In more detail, TBI patients exhibit an impaired ability to allocate attention when required to utilize top-down attentional control with a directional cue, and struggle to disengage from an incorrect spatial cue. These notable outcomes provide strong evidence for the use of these kinds of tasks as informative functional markers for attention and cognition after TBI. They may be used for monitoring recovery or tracking the efficacy of treatment and rehabilitation programs in improving cognitive function. The evidence that TBI significantly impacts visual attention is affirmed and a need for further research to systematically assess this deficit has been identified.

## Limitations

There were several limitations to this study. In particular, there were a number of sources of heterogeneity that could not be fully described. Etiology was not well-reported amongst studies and therefore was a likely source of variability. Future researchers should aim to investigate the contribution of etiology to attention deficit after TBI, particularly with growing accounts of blast-related injuries in armed combat. In addition, injury related factors such as intracranial pressure, injury to the orbit and length of in care might also be contributing factors to the heterogeneity observed in the present study, particularly with moderate and severe TBI cases. Unfortunately, such details are not usually reported in TBI studies on visual attention, and future studies may wish to consider noting the characteristics of the TBI injury.

Task design remains a significant source of variability between studies. In particular, visual search task paradigms can vary widely between studies from timed cancellation tasks to the no/neutral cue conditions of the Covert Orienting of Attention Task and Attention Network Test. A systematic assessment of all the analyzed conditions is needed to help reduce this variability and aid in the understanding of attention allocation and processing. Importantly, the reorienting process for exogenously allocated attention is in dire need of investigation as only two of the included studies involved an invalid exogenous cue condition with very different results (effect sizes of 0.63 and 2.21). Until more data is collected in this condition, it is unclear just how exogenous attention engagement and disengagement is affected following TBI.

Future research should focus on the development of a valid paradigm which includes an un-cued condition as well as valid and invalid conditions for both endogenous and exogenous attention allocation tasks. These tasks should also look at response time and accuracy as key measures to help confirm or clarify the reported distinction between these two outcome measures.

Another identified gap in the literature was the under-sampling of some age ranges and post-injury periods. Additional data to contribute to each severity group, particularly the severe subgroup, would help paint a clearer picture of the process for recovery of attention deficit after TBI. More data focusing only on moderate TBI may be useful to distinguish whether this visual attention deficit continues to degrade with increased injury severity, or if the moderate and severe TBI remain similar.

## Data Availability Statement

The original contributions generated for the study are included in the article/Supplementary Material, further inquiries can be directed to the corresponding author/s.

## Author Contributions

JW was the primary contributing author to the drafting of the manuscript. All authors contributed substantially to the conception of the work, data collection and analysis, and critical revision of the manuscript.

## Conflict of Interest

The authors declare that the research was conducted in the absence of any commercial or financial relationships that could be construed as a potential conflict of interest.
