# Supplementary material for: Visuospatial Attention Allocation as an Indicator of Cognitive Deficit in Traumatic Brain Injury: A Systematic Review and Meta-Analysis
Source: Front Hum Neurosci. 2021 Jul 20;15:675376. doi: 10.3389/fnhum.2021.675376 (PMC8329082; doi:10.3389/fnhum.2021.675376)
Supplement: Supplementary file 1 [file Table_1.DOCX]

**Appendix I**

**Summary of outcome measures from included studies used in the meta-analysis.**

| First Author | Year | Severity | Subjects | Mean Post-Injury Period (days) | Task Type | Outcome Measures | Results | | |
| --- | --- | --- | --- | --- | --- | --- | --- | --- | --- |
|  |  |  |  |  |  |  | **Task condition (PIP/Cue type/Task)** | **Mean ± SD for TBI group** | **Mean ± SD for Control group** |
| MacFlynn et al | 1984 | Mild | 45 Control  45 TBI | 1  Follow-up 42 & 180 | Visual Search | Response Time | Day 0  6 weeks  6 months | 999.30±410.80  827.90±358.00  631.0 ±154.40 | 671.40±167.70 |
| Cremona-Meteyard et al | 1992 | Moderate-to-severe | 9 Control  11 TBI | 2176 | Covert Orienting of Attention Task | Response Time | Valid cue  Invalid cue | 279±68  278±55 | 250±32  220±21 |
| Cremona-Meteyard et al | 1994 | Mild | 12 Control  9 TBI | 14  Follow up 365 & 730 | Covert Orienting of Attention Task | Response Time | 2 weeks  Valid cue  Neutral cue  Invalid cue  1 year  Valid cue  Neutral cue  Invalid cue  2 years  Valid cue  Neutral cue  Invalid cue | 234±24  226±37  275±40  219±38  222±28  258±41  220±19  235±33  268±34 | 235±24  212±24  288±44  231±45  240±28  278±44  212±24  235±24  288±44 |
| Geldmacher et al | 1997 | Severe | 21 Control  20 TBI | 92.5 | Visual Search | Q Score (accuracy and response time) | Q score 1:4  Q score 1:9  50 stimuli  100 stimuli | 0.58±0.23  0.34±0.17  0.45±0.24  0.46±0.23 | 1.22±0.32  0.77±0.26  1.02±0.36  0.96±0.37 |
| Hills et al | 1998 | Severe | 21 Control  20 TBI | 92.5 | Visual Search | Q Score (accuracy and response time) | Random figures  Structured figures  Structured letters  Random letters | 0.58±0.35  0.52±0.27  0.46±0.21  0.39±0.18 | 1.26±0.33  1.03±0.28  0.92±0.19  0.86±0.27 |
| Bate et al | 2001 | Severe | 30.2 (control)  28.9 (TBI) | 843.8 | Covert Orienting of Attention Task | Response Time | Single task  Valid cue  Neutral cue  Invalid cue  Dual task  Valid cue  Neutral cue  Invalid cue | 412.8±77.2  429.8±80.5  432.0±80.2  413.1±66.6  429.8±80.5  432.0±80.2 | 343.7±57.5  365.1±55.2  372.9±63.0  343.7±57.5  365.1±55.2  372.9±63.0 |
| Van Donkelaar et al | 2005 | Mild | 21 (control)  21 (TBI) | 1.542 | Attention Network Test | Response Time | Alerting effect  Orienting effect  Executive effect  All conditions | 549.08±133.93  515.54±93.59  475.24±34.64  570.86±135.55 | 476.92±111.34  468.27±121.03  440.60±103.27  515.29±108.11 |

PIP- Post-injury period, TBI -Traumatic brain injury, VC – Valid cue, IC – Invalid cue, LVF – Left visual field, RVF – Right visual field

| First Author | Year | Severity | Subjects | Mean Post-Injury Period (days) | Task Type | Outcome Measures | Results | | |
| --- | --- | --- | --- | --- | --- | --- | --- | --- | --- |
|  |  |  |  |  |  |  | **Task condition (PIP/Cue type/Task)** | **Mean ± SD for TBI group** | **Mean ± SD for Control group** |
| Halterman et al | 2006 | Mild | 20 Control  20 TBI | 1.542  Follow up 7, 14 & 28 | Attention Network Test | Response Time | Conflict effect  Orienting effect  Alerting effect  All conditions | 44.88±17.26  46.33±20.84  47.94±25.18  66.43±25.18 | 44.72±20.84  40.05±20.12  46.65±16.55  38.12±17.98 |
| Pavlovskaya et al | 2007 | Severe | 9 Control  21 TBI | 90 | Identification task with exogenous cue | Accuracy | VC Fraction correct  IC Fraction correct | 0.66±12  0.52±12 | 0.68±13  0.60±14 |
| Catena et al | 2009 | Mild | 20 Control  17 TBI | 1.583  Follow up 6, 14 & 28 | Attention Network Test | Response Time | 1 day  Obstacle contact  No contact  6 days  Obstacle contact  No contact  14 days  Obstacle contact  No contact  28 days  Obstacle contact  No contact | 63.20±11.34  57.86±21.76  45.13±19.66  43.33±19.66  50.17±21.16  45.02±16.12  47.27±18.36  49.31±25.22 | 38.12±17.98  38.12±17.98  44.72±20.84  44.72±20.84  40.05±20.12  40.05±20.12  46.65±16.55  46.65±16.55 |
| Sinnett et al | 2011 | Mild | 10 Control  8 TBI | 80.1 | Temporal order judgement Covert Orienting of Attention Task (with endogenous and exogenous cues) | Point of subjective simultaneity | Exogenous Cue  Endogenous Cue | 156.00±39.00    49.00±17.55 | 65.00±42.88  48.00±19.57 |
| Rodríguez-Bailón et al | 2012 | Severe | 9 Control  9 TBI | Not reported | Attention Network Test | Response Time | All conditions | 75.33±25.40 | 63.15±14.82 |
| Hill-Jarrett et al | 2015 | Moderate-to-severe | 12 Control  12 TBI | 2091 | Attention Network Test | Response Time | LVF Reaction time difference  RVF Reaction time difference | 25.68±22.90  51.46±28.41 | 37.93±28.42  44.96±39.11 |
| Schmitter-Edgecombe et al | 2015 | Moderate-to-severe | 40 Control  40 TBI | 41.2  Follow up 305 | Visual Search | Response Time | 41 days  Preattentive Present size 2  Preattentive Present size 8  Preattentive Absent size 2  Preattentive Absent size 8  Attentive Present size 2  Attentive Present size 8  Attentive Present size 2  Attentive Present size 8 | 752.79±242.57  770.55±269.76  790.10±261.36  854.92±406.07  832.10±205.89  1123.32±300.03  926.18±262.29  1892.60±710.67 | 533.35±82.16  541.26±76.75  528.61±104.44  540.40±104.44  603.72±85.70  803.25±152.99  661.79±98.31  1144.18±362.40 |

PIP- Post-injury period, TBI -Traumatic brain injury, VC – Valid cue, IC – Invalid cue, LVF – Left visual field, RVF – Right visual field

| First Author | Year | Severity | Subjects | Mean Post-Injury Period (days) | Task Type | Outcome Measures | Results | | |
| --- | --- | --- | --- | --- | --- | --- | --- | --- | --- |
|  |  |  |  |  |  |  | **Task condition (PIP/Cue type/Task)** | **Mean ± SD for TBI group** | **Mean ± SD for Control group** |
| Schmitter-Edgecombe (cont’d) |  |  |  |  |  |  | 305 days  Preattentive Present size 2  Preattentive Present size 8  Preattentive Absent size 2  Preattentive Absent size 8  Attentive Present size 2  Attentive Present size 8  Attentive Present size 2  Attentive Present size 8 | 629.68±195.44  635±49±222.13  671.70±267.84  716.25±371.80  727.58±235.10  1090.00±727.00  801.94±313.37  1622.78±1049.83 | 545.94±154.90  548.25±138.72  522.94±119.94  535.08±144.27  623.34±147.06  802.48±181.43  668.15±160.61  1052.83±258.77 |
| Robertson et al | 2017 | Moderate-to-severe | 30 Control  30 TBI | 38.7 | Visual Search & Visual Search with endogenous cue | Response Time | Endogenous cue  Present size 2  Present size 8  Absent size 2  Absent size 8  No cue  Present size 2  Present size 8  Absent size 2  Absent size 8 | 704.00±136.93  727.00±169.79  .00±147.89  874.00±230.04    810.00±158.84  1089.00±235.52  901.00±202.66  1767.00±558.68 | 564.00136.93  570.00±169.79  593.00±147.89  650.00±230.04  654.00±158.84  859.00±235.52  698.00±202.66  1232.00±558.68 |
| Shah et al | 2017 | Mixed | 24 Control  13 TBI | 745.38 | Attention Network Test | Response Time | All conditions | 67.84±18.36 | 58.50±16.55 |

PIP- Post-injury period, TBI -Traumatic brain injury, VC – Valid cue, IC – Invalid cue, LVF – Left visual field, RVF – Right visual field
